# Improving individualized prescription in patients with multimorbidity through medication review

**DOI:** 10.1186/s12877-022-03107-2

**Published:** 2022-05-12

**Authors:** Núria Molist-Brunet, Daniel Sevilla-Sánchez, Emma Puigoriol-Juvanteny, Matilde Barneto-Soto, Javier González-Bueno, Joan Espaulella-Panicot

**Affiliations:** 1 Department of Geriatrics, Hospital Universitari de La Santa Creu de Vic, Vic, Spain; 2 Central Catalonia Chronicity Research Group (C3RG), Centre for Health and Social Care Research (CESS), University of Vic – Central University of Catalonia (UVIC-UCC), Vic, Barcelona Spain; 3 Department of Pharmacy, Parc Sanitari Pere Virgili, Vic, Barcelona Spain; 4 Department of Epidemiology, Hospital Universitari de Vic, Vic, Barcelona Spain; 5 Laboratory of Tissue Repair and Regeneration (TR2Lab), University of Vic – Central University of Catalonia (UVIC-UCC), Fundació Hospital Universitari de La Santa Creu de Vic, and Hospital Universitari de Vic, 08500 Vic, Barcelona Spain; 6 Department of Pharmacy, Consorci Hospitalari de Vic, Vic, Barcelona Spain

**Keywords:** Medication review, Inappropriate prescription, Polypharmacy

## Abstract

**Background:**

Older patients tend to have multimorbidity, represented by multiple chronic diseases or geriatric conditions, which leads to a growing number of prescribed medications. As a result, pharmacological prescription has become a major concern because of the increased difficulties to ensure appropriate prescription in older adults. The study’s main objectives were to characterize a cohort of older adults with multimorbidity, carry out a medication review and compare the pharmacological data before and after the medication review globally and according to the frailty index.

**Methods:**

This was a quasi-experimental (uncontrolled pre-post) study with a cohort of patients ≥ 65 years old with multimorbidity. Data were collected from June 2019 to October 2020. Variables assessed included demographic, clinical, and pharmacological data, degree of frailty (Frail-VIG index), medication regimen complexity index, anticholinergic and or sedative burden index, and monthly drug expenditure. Finally, a medication review was carried out by an interdisciplinary team (primary care team and a consultant team with a geriatrician and a clinical pharmacist) by applying the Patient-Centered Prescription model to align the treatment with care goals.

**Results:**

Four hundred twenty-eight patients were recruited [66.6% women; mean age 85.5 (SD 7.67)]. The mean frail index was 0.39 (SD 0.13), corresponding with moderate frailty. Up to 90% of patients presented at least one inappropriate prescription, and the mean of inappropriate prescriptions per patient was 3.14 (SD 2.27). At the three-month follow-up [mortality of 17.7% (*n* = 76)], the mean chronic medications per patient decreased by 17.96%, varying from 8.13 (SD 3.87) to 6.67 (SD 3.72) (*p* < 0.001). The medication regimen complexity index decreased by 19.03%, from 31.0 (SD 16.2) to 25.1 (SD 15.1), and the drug burden index mean decreased by 8.40%, from 1.19 (SD 0.82) to 1.09 (SD 0.82) (*p* < 0.001). A decrease in polypharmacy, medication regimen complexity index, and drug burden index was more frequent among frail patients, especially those with severe frailty (*p* < 0.001).

**Conclusions:**

An individualized medication review in frail older patients, applying the Patient-Centered Prescription model, decreases pharmacological parameters related to adverse drug effects, such as polypharmacy, therapeutical complexity, and anticholinergic and, or sedative burden. The benefits are for patients with frailty.

## Background

Pharmacological prescription in older adults has become a worldwide concern because of the growing number of prescribed medications [1] and increased difficulties guaranteeing appropriate prescription based on each patient’s profile [2]. Evidence shows that the use of medicines in older adults is often inappropriate [3, 4].

Older adults tend to have multimorbidity, represented by multiple chronic diseases or geriatric conditions due to organ changes caused by aging, which require a variety of medical management [5]. This profile of patients often present criteria for frailty [3], a common clinical syndrome in older adults that carries an increased risk of poor health outcomes [6].

Moreover, patients with multimorbidity tend to have polypharmacy [1], considered when a patient is taking five or more medications continuously [7], which is a risk for adverse drug events (ADE) [4, 8]. Polypharmacy, and especially severe or excessive polypharmacy (ten or more chronic medications [7]), has been associated with hospital admissions, functional impairment, higher mortality, and also increased healthcare expenditure [4]. As it is known, polypharmacy is a warning sign of inappropriate prescription (IP) [8]. The definition of IP includes prescribing medications with a higher risk for an ADE than its clinical benefit. Other causes of IP can be a medication used longer than indicated, with a higher frequency or duplicity, drug-drug or drug-disease interaction, or finally, if an indicated medication is not prescribed [9]. IP is associated with negative health outcomes, such as ADE, hospital admissions, redundant use of health services and, ultimately, death [2].

Globally, it is known that frail patients with polypharmacy are at higher risk of ADE than non-frail patients, although they present polypharmacy as well [10]. Additionally, it should be noted that polypharmacy is also a risk factor for suboptimal adherence to pharmacological treatment [11], and low adherence may lead to increased morbidity, mortality, and cost [12].

Because of the global increase in older frail patients with multimorbidity, it becomes mandatory to elaborate a methodology to optimize prescription [5]. A multidimensional approach, including physical and cognitive function, social characteristics, and disease severity with a global assessment of health needs and priorities, could reduce the burden of adverse drug events in older adults [5, 13]. Consequently, there should be differences in deciding treatment and prescribing drugs based on the patient’s baseline status.

Overall, it is accepted that frail older patients with polypharmacy need a periodic medication review (MR) by means of a specific tool which considers parameters such as quality of life, functionality, main care goal, and life expectancy [14]. The MR will enable to detect drug-related problems and recommend interventions to guarantee individualized prescription. The Pharmaceutical Care Network Europe (PCNE) has defined three types of MR (structural evaluation of the patient’s medicines to optimize their use and improve health outcomes): simple, intermediate, and advanced [15]. In this context, the Patient-Centered Prescription Model (PCP) [16] could be considered an advanced MR (based on medication history, patient information, and clinical information) [15, 17]. Different studies applying the PCP model show the capacity to identify IP, optimize the polypharmacy, and the improvement of the medication adherence in different profiles of older patients [18–22].

### Objectives of the study

The main objectives of the study were: i) to characterize a cohort of older adults with multimorbidity, analyze their baseline situation and calculate their frailty index (FI); ii) to carry out an MR through the application of the PCP model and compare the pharmacological data (polypharmacy, medication regimen complexity and anticholinergic and or sedative burden) before and after the MR, globally and according to FI; and iii) to analyze monthly medication expenditure before and after the MR.

## Methods

### Design and participants

The study has a quasi-experimental design (pre-post study) including a cohort of patients with multimorbidity (Community Older Patients cohort (COP cohort)) [16, 23], in the Osona County, a mixed urban–rural district from Catalonia (Spain), with a three-month follow-up. Data were collected from June 2019 to October 2020.

Inclusion criteria: patients aged 65 or older with multimorbidity (two or more morbidities) in whom their General Practitioner identified difficulties with prescription management and the need for an MR by a consultant team (geriatrician and a clinical pharmacist).

Exclusion criteria: patients in their probable last hours or days of life [24].

Ethics approval: The study was approved by the different local Scientific Ethics Committee, according to the place where patients received care: 1) FORES (Fundació d’Osona per la Recerca i l’Educació Sanitàries), under reference number 2019–106/PR237; 2) IDIAP Jordi Gol, under reference number 19/206-P. 3) Fundació Catalana d’Hospitals, under reference number CEI 20/23. We obtained informed consent from the patient, or in cases of incapacity, from the main caregiver. Afterward, we included patients’ informed consent in their electronic health records.

### Data collection

Data were collected at the beginning of the study, before the MR, and after three months.

- Personal data: Age and sex were recorded.
- Functional data: i) Dependence/independence for medication management (only patients who lived in their own home were considered, given that medication self-management is unusual in a nursing home); ii) the Barthel Index (BI) was used to assess basic activities of daily living [25, 26]
- Medical data: i) Morbidities (from expanded diagnostic clusters within the Johns Hopkins University ACG system) [27] and adjusted-age Charlson Index [28]. As other morbidities, we considered that a patient suffered depressive syndrome when it was collected in medical records or when they took a specific medication treatment; ii) dementia diagnosis, as stated in the medical records, and the degree of deterioration according to the GDS (Global Deterioration Scale) [29]; iii) blood pressure levels measured in the last year; iv) geriatric syndromes.
- Analytical data: i) Full blood count; ii) urea; iii) electrolytes; iv) glycosylated hemoglobin (HbA1c) if available in the last year.
- Frailty index (FI): It was measured using the Frail-VIG index [30]. FI was categorized as: i) FI < 0.20: no frailty; ii) FI 0.20–0.35: mild frailty; iii) FI 0.36–0.50: moderate frailty iv) FI > 0.50: severe frailty.
- Pharmacological data: The number of chronic medications was recorded for at least six months before the MR (baseline) and after the three-month follow-up (post-MR). Polypharmacy was analyzed at baseline and post-review, and it was categorized in three different degrees according to the number of medications: i) 0–4 medications: no polypharmacy; ii) from 5 to 9 medications: moderate polypharmacy; and iii) 10 or more medications: excessive polypharmacy [7]. After a 3-month follow-up, pre and post polypharmacy degrees were compared to identify differences in polypharmacy degree due to MR (a decreased, unaltered, or increased degree was identified).
- Medication Regimen Complexity Index (MRCI) [31, 32]: It was analyzed at baseline and post-MR, and it was categorized into three different degrees according to the result: i) 0–19.99: low complexity; ii) 20–39.99: moderate complexity; iii) high complexity if ≥ 40. Pre- and post-MRCI degrees, measured at baseline and the three-month follow-up, respectively, were also compared to assess any increase, maintenance, or decrease due to the MR carried out.
- Anticholinergic and or sedative burden [33]: It was assessed using the drug burden index (DBI). It was analyzed at baseline and post-MR, and the DBI was categorized into three different degrees according to the result: i) 0–0.99: low; ii) 1–1.99: moderate; iii) ≥ 2: high anticholinergic and or sedative burden. At the three-month follow-up, pre and post-DBI degrees were also compared.
- Identification of end-of-life patients (EOL patients) (using NECPAL CCOMS-ICO© tool criteria) [34]: Patients identified as being in their last months or year of life. The criteria to identify them as EOL patients were based on: i) their primary care physician; ii) advanced illness criteria [34]; or iii) Frail-VIG index > 0.50.
- Main therapeutic goal: It was established through consensus with the patient, their usual healthcare team and the consultant team taking into account their baseline situation [35]: i) survival in patients with a fit baseline situation; ii) functional status in patients with an intermediate situation; iii) symptomatic in the most vulnerable patients (EOL patients were considered to be included). The therapeutic goal was established by consensus with the patient, the usual healthcare team, and the consultant team [35].
- Mortality: At the three-month follow-up and with the total number of patients alive, the comparative study between pre and post-pharmacological data was carried out.
- Monthly drug expenditure (MDE): The cost of prescribed medications for each patient was collected (in euros (€)). Monthly expenditure vs. frailty degrees was also analyzed.

### Medication review

**Fig. 1:**
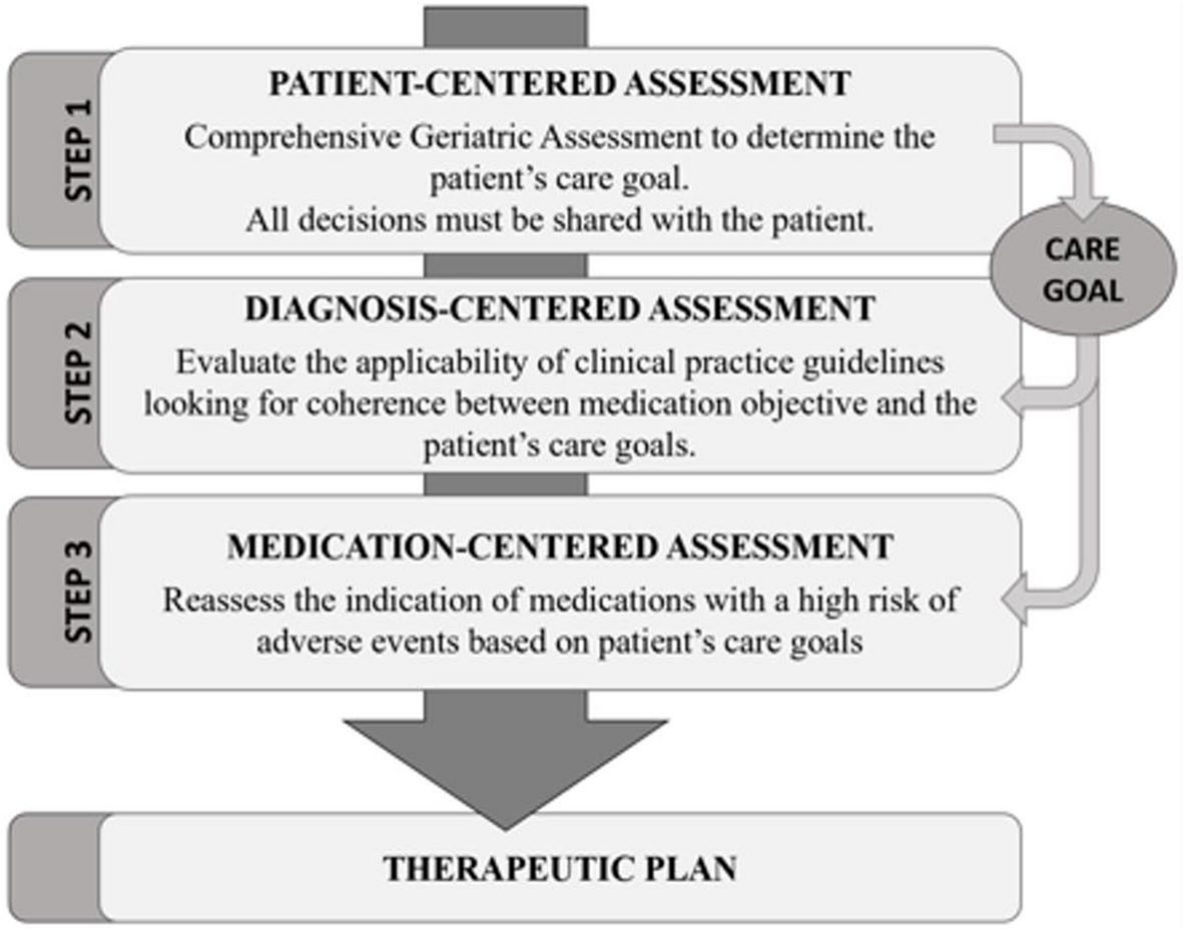
Patient-centered prescription model (PCP model)

Each patient’s treatment was analyzed by applying the PCP model. This model is a systematic process with four stages carried out by an interdisciplinary team (the patient’s General Practitioner and nurse with a consultant team (performed by a geriatrician and a clinical pharmacist). In this model the therapeutic decisions are based on the patient’s global assessment (Comprehensive geriatric assessment (CGA), calculation of the frailty index (Frail-VIG index) [36]), and the resulting individual therapeutic goal (prolonging survival, maintaining functionality, or prioritizing symptomatic control) [37]. In incapacity cases, the decisions were made with the main caregiver (Fig. 1). The interdisciplinary team held periodic meetings to carry out the MR of the patients who were identified by the General Practitioner and consented to participate and who gave their consent. These meetings took place in three different Primary Care Centers and in three different nursing homes of the same area.

### Criteria used to determine inappropriate prescriptions

An MR was carried out. Based on the current evidence, different criteria were used to determine inappropriate prescriptions in the most prevalent chronic conditions [23]: type 2 Diabetes Mellitus (T2DM) (guidelines of the American Diabetes Association (ADA) were applied [38, 39]); hypertension and cardiovascular therapy [40]; dyslipidemia [41]; mental health and dementia (the recommendations of the European Association of Palliative Care were followed [42] and, the progressive dose decrease of chronic antipsychotic treatment was proposed in individuals who had not suffered behavioral disorders in the last 3–6 months [40, 43]); and chronic pain (following Beers and STOPP criteria [44–46]). The STOPPFrail criteria were applied in patients identified at the end of life (according to NECPAL CCOMS-ICO© [34]) [40].

### Sample size

For sample size calculation, IP in the overall older frail population was estimated at 71% [47]. With a 95% confidence level and 5% accuracy, a minimum of 352 patients should be included.

### Data analysis

Statistical analysis was performed with the IBM SPSS Statistics v27.0 statistical software. The results for categorical variables were expressed as absolute and relative frequencies, and results for continuous variables were analyzed using both parametric and non-parametric statistics, depending on the level and distribution of data (as means and standard deviations (SD) or median, Q1 and Q3 and minimum and maximum values). The Chi-Square test (or Fisher’s exact test in 2 × 2 tables where the expected frequencies were lower than 5) was used for categorical variables, and Student’s t-test to analyze the relationship between quantitative and categorical variables following the normal distribution, and the Mann–Whitney U when variables did not follow it. Statistical test for paired data: McNemar test for categorical variables or the T-Student for paired data for quantitative variables following the normal distribution or Wilcoxon Test for quantitative variables that did not follow it was used to analyze the impact of the intervention. Statistical significance was considered when the value of p was lower than 0.05.

## Results

Initially, 428 patients were included (285 (66.6%) were women). The mean age was 85.5 years (SD 7.67). Demographic, clinical, and pharmacological baseline data are listed in Table 1. Globally, they had moderate dependence for basic daily activities, with a mean Barthel Index of 49.93 (SD 32.14), and 316 (73.8%) of them suffered some degree of dementia. Up to 46.3% suffered depressive syndrome. The mean frail index was 0.39 (SD 0.13), corresponding to moderate frailty. 36.2% of the subjects were identified as being in an end-of-life situation, and only 57 (27.1%) patients managed their medication.

**Table 1:** Baseline data

Baseline data (*n* = 428)		Mean [sd]	Frequency (%)
**Demographic data**			
Age,mean [sd]		85.52 (7.67)	
Sex	Men		143 (33.4%)
	Women		285 (66.6%)
Origin	Home		210 (49.1%)
	Nursing Home		218 (50.9%)
**Clinical, functional, and cognitive data**			
Medication management (only patients living at home) (*n* = 210)			57 (27.1%)
Barthel Index, mean [sd]			49.93 (32.14)
Barthel Index	Independence: BI ≥ 95		51 (11.9%)
	Mild dependence: BI 90–65		120 (28.0%)
	Moderate dependence: BI 60–25		129 (30.2%)
	Severe dependence: BI ≤ 20		128 (29.9%)
Cognitive status	No dementia		112 (26.2%)
	Mild dementia		62 (14.5%)
	Moderate dementia (from GDS 5 to GDS 6B)		112 (26.2%)
	Advanced dementia (from GDS 6C)		142 (33.1%)
Geriatric syndromes, mean [sd]		2.92 (1.52)	
Type of geriatric syndrome	Falls		144 (33.6%)
	Dysphagia		84 (19.6%)
	Pain		99 (23.1%)
	Depressive syndrome		198 (46.3%)
	Insomnia		229 (53.5%)
Morbidities, mean [sd]		4.91 (2.16)	
Most frequent morbidities	Hypertension		290 (67.8%)
	Chronic renal failure		186 (43.5%)
	T2DM		110 (25.7%)
	Heart Failure		88 (20.6%)
Morbidities	1–2		43 (10.0%)
	3–4		168 (39.3%)
	5 or more		217 (50.7%)
Charlson Index, mean [sd]		3.26 (2.27)	
Frailty index, mean [sd]		0.39 (0.13)	
Frailty index, degrees	No frailty (0–0.19)		32 (7.5%)
	Mild frailty (0.20–0.35)		113 (26.4%)
	Moderate frailty (0.36–0.50)		201 (47.0%)
	Severe frailty (> 0.50)		82 (19.1%)
End-of-life patients			155 (36.2%)
Therapeutic goal	Survival		41 (9.6%)
	Functionality		223 (52.1%)
	Symptomatic		164 (38.3%)
**Pharmacological data**			
Number of chronic medications, mean [sd]		8.13 (3.88)	
Polypharmacy, degree	0–4 medications		80 (18.7%)
	5–9 medications		205 (47.9%)
	10 or more medications		143 (33.4%)
MRCI, mean [sd]		30.74 (16.26)	
MRCI, degree	Low complexity (0–19.99)		109 (25.5%)
	Moderate complexity (20–39.99)		208 (48.6%)
	High complexity (40 or more)		111 (25.9%)
DBI, mean [sd]		1.17 (0.84)	
DBI, degree	Low DBI (0–0.99)		70 (16.4%)
	Moderate DBI (1–1.99)		197 (46.0%)
	High DBI (2 or more)		161 (37.6%)
Inappropriate Prescriptions (IP), mean [sd]		3.14 (2.27)	
IP	0 IP		43 (10.0%)
	1 or more IP		385 (90.0%)
	2 or more IP		322 (75.2%)
	3 or more IP		246 (57.5%)

*Abbreviations***:** *Sd* Standard deviation, *BI* Barthel index, *GDS* Geriatric syndromes, *T2DM* Type 2 diabetes mellitus, *MR* Medication review, *MRCI* Medication regimen complexity index, *DBI* Drug burden index

Regarding IP detection, up to 90% of the patients presented at least one IP, and the mean IP per patient was 3.14 (SD 2.27).

At the three-month follow-up, up to 76 patients had died (17.7%). Therefore, in the comparative study pre and post, the pharmacological data from the 352 patients were analyzed at three months. A total of 1334 proposals to optimize medication were made (proposals of medication withdrawal (812 (60.9%)), dose adjustment (386 (28.9%)), or indication of a new medication (136 (10.2%)). After three months, 864 proposals were implemented, which accounted for 64.7% of the initial proposals. There were different reasons why proposals were not implemented, such as disagreement with the patient’s or the primary care team’s criteria or difficulties with withdrawal tolerance (especially with psychotropic medications).

Pre and post-pharmacological data were compared based on sex and frailty degree (Table 2). After the three-month follow-up, the mean chronic medications per patient decreased by 17.96%, from 8.13 (SD 3.87) to 6.67 (SD 3.72) (*p* < 0.001). MRCI and DBI scores decreased significantly. The mean MRCI decreased by 19.03%, from 31.0 (SD 16.2) to 25.1 (SD 15.1) (*p* < 0.001), and the mean DBI decreased by 8.40%, from 1.19 (SD 0.82) to 1.09 (SD 0.82) (*p* < 0.001).

**Table 2:** Pre-post MR analysis of medication number, MRCI, DBI and MDE

Pharmacological data		Previous MR	Post-MR	Difference	P
**Number of medications, mean [sd]**					
All patients		8.13 (3.94)	6.67 (3.72)	-1,45 (1,97)	< 0.001
Sex	Men	8.43 (3.57)	6.98 (3.68)	-1,45 (1,94)	< 0.001
	Women	7.95 (4.10)	6.50 (3.74)	-1,45 (1,99)	< 0.001
Frailty degree	No frailty: 0–0.19	7.19 (4.50)	6.65 (3.85)	-0,54 (2,34)	> 0.05
	Mild frailty: 0.20–0.35	7.67 (3.48)	6.79 (3.27)	-0,88 (1,82)	< 0.001
	Moderate frailty: 0.36–0.50	8.18 (4.16)	6.79 (4.19)	-1,40 (1,61)	< 0.001
	Severe frailty: > 0.50	9.18 (3.59)	6.05 (2.90)	-3,13 (2,12)	< 0.001
**Medication regimen complexity index, mean [sd]**					
Whole sample		31.0 (16.2)	25.1 (15.1)	-5.9 (8.2)	< 0.001
Sex	Men	31.3 (14.4)	25.0 (14.3)	-6.3 (7.6)	< 0.001
	Women	30.9 (17.1)	25.2 (15.4)	-5.7 (8.5)	< 0.001
Frailty degree	No frailty: 0–0.19	27.2 (18.6)	25.6 (15.4)	-1.6 (10.5)	> 0.05
	Mild frailty: 0.20–0.35	28.7 (14.1)	24.8 (13.1)	-3.9 (7.7)	0.002
	Moderate frailty: 0.36–0.50	31.6 (17.4)	25.9 (17.1)	-5.7 (6.6)	< 0.001
	Severe frailty: > 0.50	35.5 (14.5)	23.1 (11.9)	-12.4 (9.1)	< 0.001
**Drug burden index, mean [sd]**					
Whole sample		1.19 (0.82)	1.09 (0.82)	-0.10 (0.35)	< 0.001
Sex	Men	1.14 (0.90)	1.00 (0.86)	-0.14 (0.38)	< 0.001
	Women	1.22 (0.78)	1.13 (0.80)	-0.09 (0.34)	< 0.001
Frailty degree	No frailty: 0–0.19	0.47 (0.53)	0.45 (0.44)	-0.02 (0.28)	0.794
	Mild frailty: 0.20–0.35	0.84 (0.65)	0.78 (0.65)	-0.06 (0.32)	0.083
	Moderate frailty: 0.36–0.50	1.40 (0.84)	1.27 (0.89)	-0.13 (0.38)	< 0.001
	Severe frailty: > 0.50	1.58 (0.73)	1.43 (0.72)	-0.15 (0.33)	0.002
**Monthly drug expenditure, median (Q1;Q3)**					
All patients		57.61 (27.01;116.54)	45.72 (20.32;99.26)	-4.81 (-16.5;0)	< 0.001
Sex	Men	78.69 (33.71;135.32)	66.19 (25.08;121.42)	-5.53 (-19.1;0)	< 0.001
	Women	50.59 (23.06;107.00)	41.55 (18.36;93.37)	-4.72 (-13.9;0)	< 0.001
Frailty degree	No frailty: 0–0.19	32.48 (15.89;84.40)	49.29 (11.32;85.87)	-0.59 (-4.5;8.8)	0.935
	Mild frailty: 0.20–0.35	50.07 (21.63;100.11)	43.45 (19.90;99.26)	-2.14 (-9.98;0)	0.040
	Moderate frailty: 0.36–0.50	68.77 (27.90;121.13)	48.17 (20.25;107.97)	-5.97 (-16.9;0)	< 0.001
	Severe frailty: > 0.50	63.21 (38.25;137.53)	47.86 (25.93;93.06)	-11.3(-22.33;3.69)	< 0.001

*Abbreviations*: *Sd* Standard deviation, *MR* Medication review

MDE was also calculated. A decrease in expenditure was detected (17.17%) after the MR, from a median of 57.61 € (Q1 27.01; Q3 116.54) to 47.72 € (Q1 20.32; Q3 99.26) (*p* < 0.001). Based on sex, there were no differences in MDE. On the other hand, regarding frailty, there was a higher decrease in monthly expenditure secondary to the MR in patients with higher frailty (*p* < 0.001) (Table 2) due to a higher number of withdrawn medications.

**Fig. 2:**
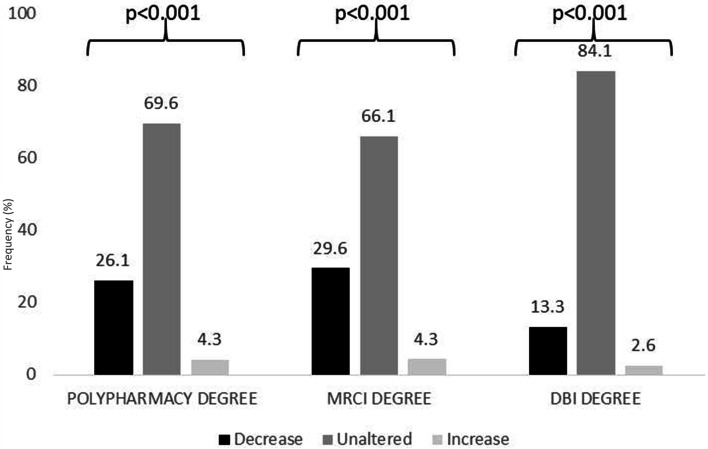
Modifications in polypharmacy, Medication Regimen Complexity Index (MRCI), and anticholinergic and or sedative burden (DBI) degrees after the medication review (MR)

Figure 2 shows polypharmacy, MRCI, and DBI degrees modifications after applying the PCP model. Regarding polypharmacy, 26.1% of patients presented a decrease of one or two degrees, and only 4.3% increased their polypharmacy degree. 29.6% of patients showed a decrease of one or two categories of their MRCI, with an increase of 4.3%. Finally, concerning the DBI degree, up to 13.3% of patients presented a decrease of one or two degrees, and 2.6% had an increase in the DBI degree (*p* < 0.001).

**Fig. 3:**
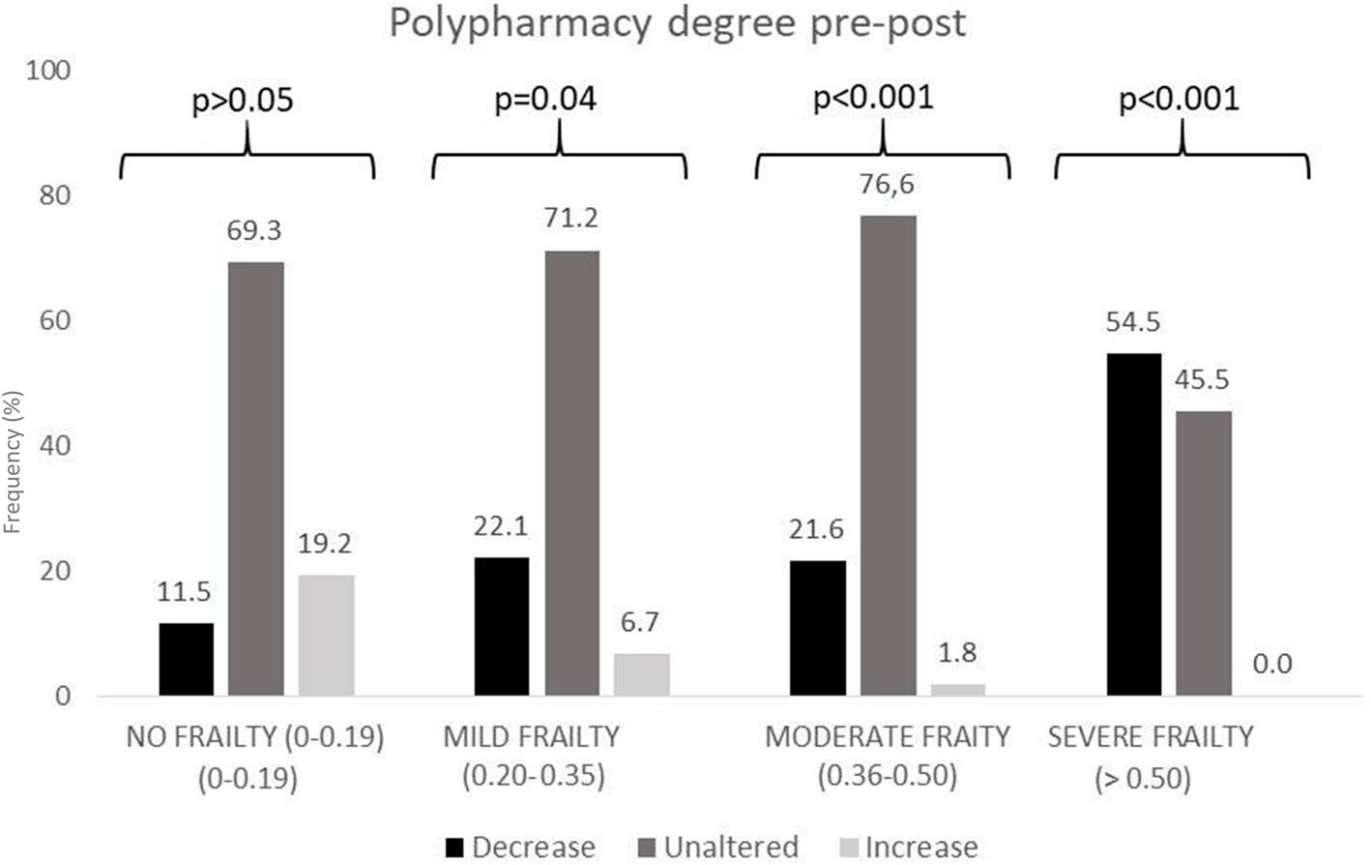
Modifications in polypharmacy degree after medication review (MR) according to VIG-Frail index

**Fig. 4:**
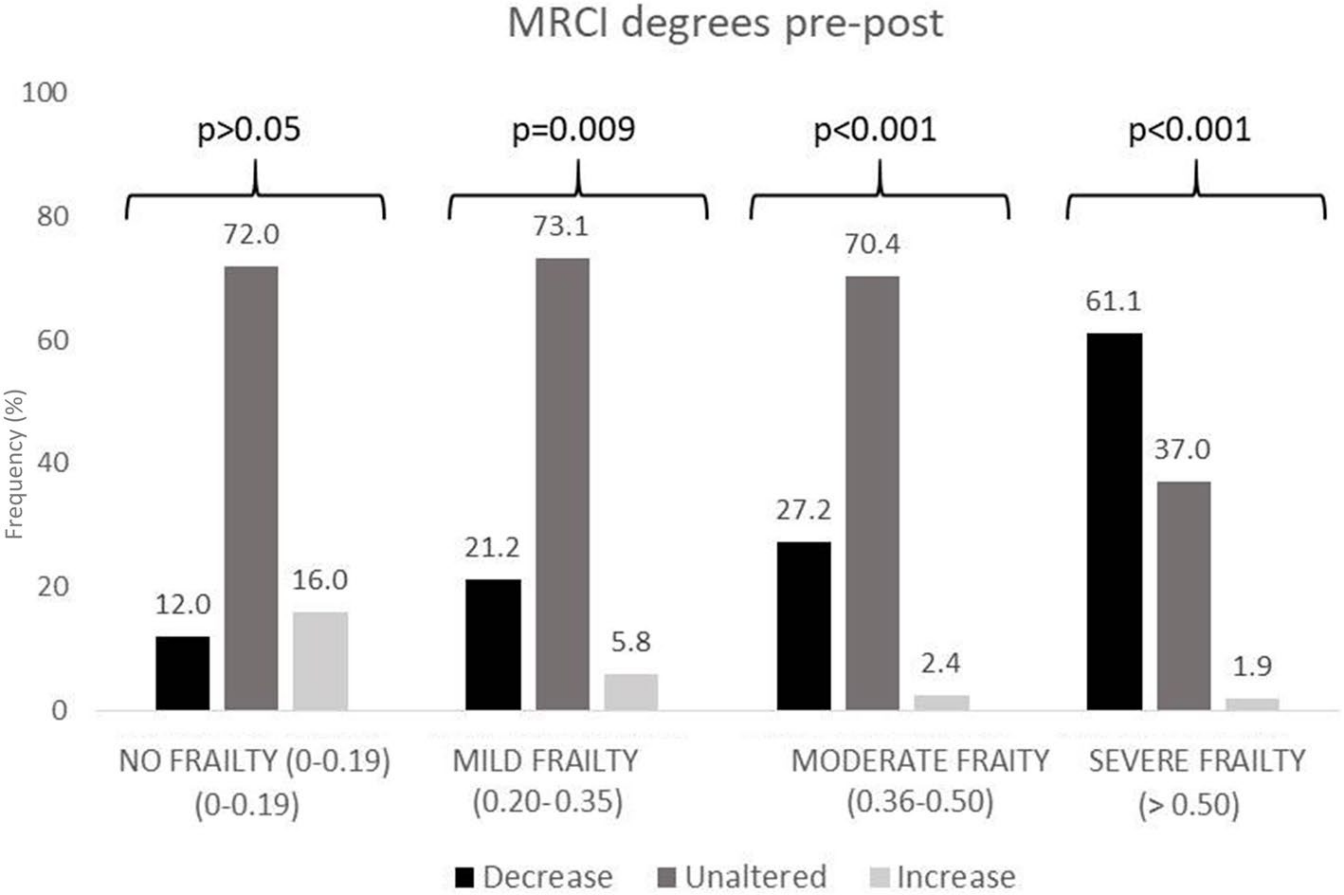
Modifications in medication regimen complexity index (MRCI) degree after medication review (MR), according to VIG-Frail index

**Fig. 5:**
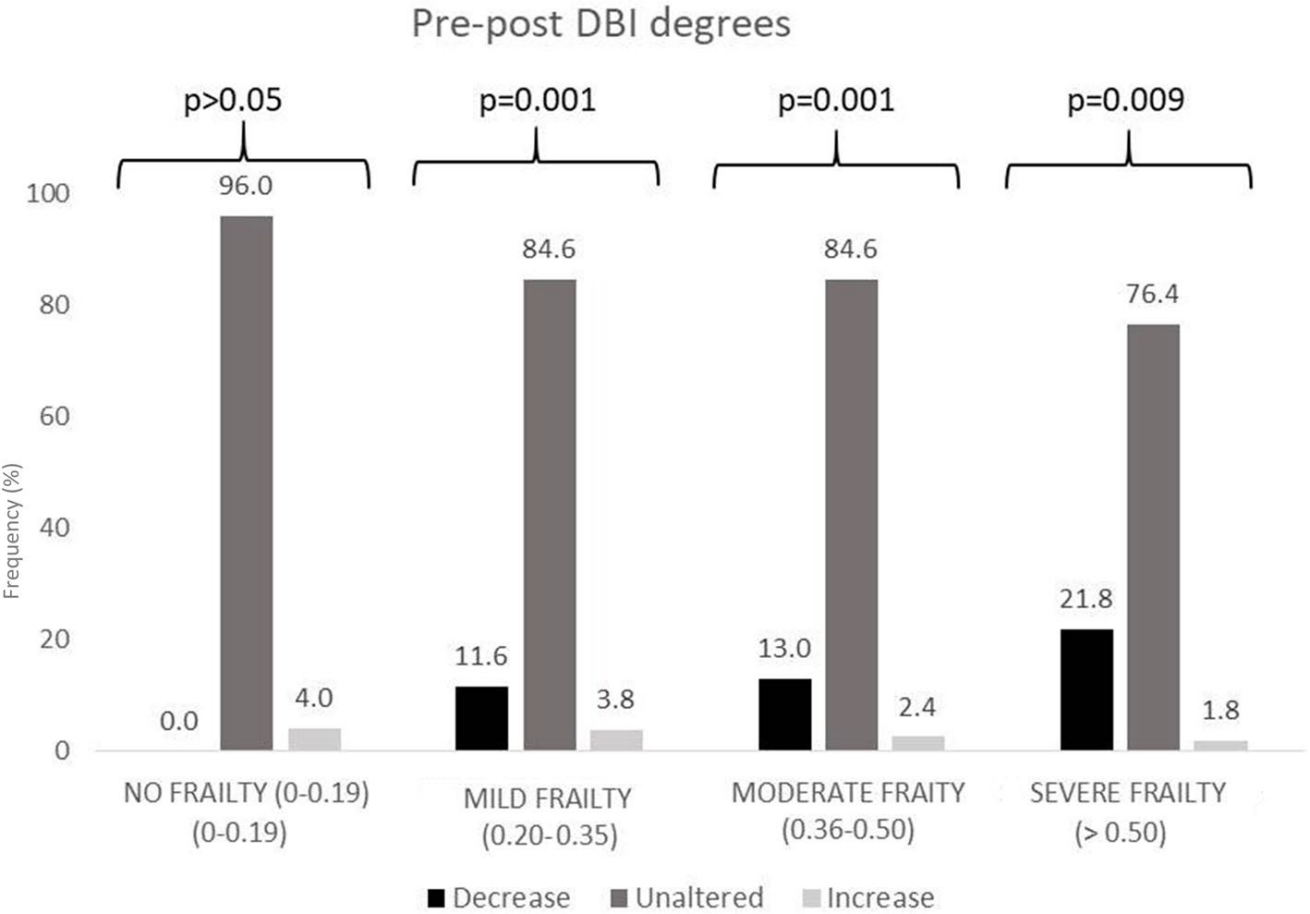
Modifications in anticholinergic and or sedative burden (DBI) degree after the medication review (MR), according to the VIG-Frail index

Figures 3, 4 and 5 show polypharmacy, MRCI, and DBI degree variation after the MR, based on frailty degree. A decrease of one or two degrees in polypharmacy, MRCI, and DBI classification was more frequent among frail patients, especially patients with severe frailty (*p* < 0.001). Moreover, in non-frail patients, there was a small number of modifications in these degrees.

Table 3 shows the types of IP analyzed and the proposals applied to optimize medication after the three-month follow-up, according to the Anatomical, Therapeutic, and Chemical (ATC) classification. It is essential to highlight that the groups with the most frequently applied proposals were: ATC A (alimentary tract and metabolism), B (blood and blood-forming organs), and C (cardiovascular system), with a percentage of 70.3%, 71.0%, and 64.8% respectively. On the other hand, the ATC group with less frequently applied proposals was N (nervous system), with a percentage of 56.8%. Note that these four ATC groups (A, B, C, and N) accounted for up to 90% of applied proposals. We did not consider ATC groups with five or less of the total proposals.

**Table 3:** Types of IP based on the Anatomical, Therapeutic, and Chemical (ATC) classification

ATC group	Number of IP identified	Number of proposals applied	% of proposals applied
A–Alimentary tract and metabolism	330 (24.7%)	232	70.3%
B–Blood and blood-forming organs	124 (9.3%)	88	71.0%
C–Cardiovascular system	412 (30.8%)	267	64.8%
D–Dermatological	0	0	0
G–Genitourinary system and hormones	26 (1.9%)	17	65.4%
H–Systemic hormonal preparations (Excluding sex hormones and insulin)	7 (0.5%)	4	57.1%
J–Anti-infective for systemic use	1 (0.1%)	0	100%
L–Antineoplastic and immunomodulation agents	2 (0.2%)	2	100%
M–Musculoskeletal system	28 (2.3%)	17	60.7%
N–Nervous system	329 (24.6%)	187	56.8%
R–Respiratory system	69 (5.1%)	46	66.7%
S–Sensory organs	6 (0.4%)	4	66.7%
V–Various	0	0	0
TOTAL	1334	864	

## Discussion

The study identified up to 90% of IP in a sample of older patients with multimorbidity by applying a personalized MR focused on an individualized therapeutic goal. Moreover, it showed a significant improvement in pharmacological outcomes, such as a 17.96% decrease in the mean of chronic medications per patient, a 19.03% decrease in MRCI mean and an 8.40% decrease in DBI mean. All these results were particularly relevant in the frailest patients.

Globally, there was a significant number of patients with an improvement in the degree of polypharmacy (26.1%), MRCI (29.6%), and DBI (13.3%) (*p* < 0.001).

The finding that only 27.1% of patients self-manage their medication might be partially related to the high therapeutic complexity of their pharmacological treatment. Medication burden, assessed through the MRCI, is a well-known factor negatively associated with medication adherence in older patients with multimorbidity and polypharmacy [48]. Therefore, interventions aimed to improve effective prescribing might enhance patient autonomy for medication management by reducing their medication burden.

Mortality at the three-month follow-up was higher than expected. This fact can be explained because data were collected during the COVID-19 pandemic when a high proportion of patients died secondarily to this disease [49].

The PCP methodology enabled us to identify a high number of patients with at least one IP (90.0%). It is much higher than the IP proportion detected in other studies with older patients [50]. The inclusion criteria could explain this, because older individuals with multimorbidity are at high risk of IP [51]. Indeed, some studies applying explicit criteria, such as STOPP-START criteria, detected a similar prevalence of IP when they selected a sample of people with multimorbidity [52].

Following current recommendations [53, 54], the PCP model aligns the treatment with care goals. As a result, significant reductions in the mean number of chronic medications and the prevalence of patients exposed to polypharmacy were detected, especially in frail patients.

Furthermore, the study showed that a prescription focused on the individualized main therapeutic goal directly impacts the different measures associated with the risk of adverse effects beyond polypharmacy, such as therapeutical complexity and anticholinergic and or sedative burden, with significant reductions. Thus, the PCP model entails both quantitative and qualitative optimization. In addition, it should not be overlooked that the application of the PCP model resulted in a decrease in MDE (17.17%).

The medications more frequently prescribed inappropriately were similar than for other studies [55]. It is remarkable that Nervous System medications were quite frequently identified as IP but, they had the lowest proportion of applied proposals. A reason for this could be that this group of drugs is intended as a symptomatic objective, resulting in great barriers from physicians to modify them and from patients to accept them.

Considering the influence of medication burden on patient’s adherence [56], the reduction in the number of chronic prescriptions, prevalence of polypharmacy, and MRCI allows us to expect a benefit from our intervention in medication adherence by non-institutionalized patients [22]. A medication adherence improvement derived from the application of the PCP model has already been shown in patients with multimorbidity [22].

Given these relevant findings and their prolongation in time, it is important to analyze the underlying reasons. First, the inclusion criteria could have played a significant role; another reason is probably the methodology, which includes personalized prescription focused on the main therapeutic individual goal; in third place, it is essential to highlight that the decisions were shared with the patient or main caregiver, and it presumably helps maintain the prescription proposals. Moreover, the proposed work team (combining the usual healthcare team with a consultant team) could also have facilitated setting the prescription proposals.

The current study had some limitations, such as the lack of a higher number of non-frail patients, which would have allowed us to carry out a more accurate statistical analysis. A further limitation was the lack of clinical outcomes in the analysis. Furthermore, the expenditure analysis did not consider the cost of the professionals dedicated to optimizing medication. Another limitation is that medication adherence was not analyzed in this cohort. Finally, it could be of interest to analyze the reasons for the rejection of optimizing proposals (which was not the study goal) in a future study.

In addition, a possible confounding factor should be highlighted: the study showed that the greater the frailty, the greater the DBI and MRCI. Therefore, in view of the high mortality observed, is it possible that patients with higher DBI and MRCI have died; in that case, the benefits of the intervention could be magnified.

It is important to consider the non-response bias in cases of patients who were initially proposed by the primary care team and later were discussed during the meeting with the consultant team, but who finally did not keep in touch with the primary care team, and for whom no proposals could be applied. At the three-month follow-up, these cases were analyzed as cases with no proposals accepted. Thus, they could have led to an underestimation of the results.

Another issue to consider is the representativeness of the sample; as a quasi-experimental study without a random selection of patients, this representativeness could be disturbed, but general data about our sample showed results consistent with other studies with frail patients [57]. Concerning the generalization of the study results, it should be note that PCP Model is a moderately established practice in the studied area [18–22]; therefore, the baseline pharmacological data could be considerably better at the beginning of the study than the baseline pharmacological data of other territories where an individualized MR is not established.Globally, the study suggests that an individualized MR is well accepted by patients and, can lead to substantial benefits for pharmacological outcomes. However, it would be significant in a future study to evaluate the impact of an individualized MR on clinical outcomes such as frailty status, falls, or cognition.

## Conclusions

An individualized medication review in older patients with multimorbidity, applying the PCP model, could lead to improved pharmacological parameters related to adverse drug effects, such as polypharmacy, therapeutical complexity, and anticholinergic and or sedative burden. The most relevant benefit would be for patients with frailty. Prospective studies with a robust design (i.e.: cluster random control trial) should be performed to demonstrate this quasi-experimental study.

## Data Availability

The datasets generated and/or analyzed during this study are available from the corresponding author on reasonable request.

## Abbreviations

ADA: American Diabetes Association
ADE: Adverse Drug Effects
BI: Barthel Index
CGA: Comprehensive Geriatric Assessment
COP: Community Older Patients
DBI: Drug Burden Index
EOL: End-Of-Life
FI: Frailty Index
FORES: Fundació d’Osona per la Recerca Educació Sanitàries
GDS: Global Deterioration Scale
GFR: Glomerular Filtration Rate
HbA1c: Glycosylated Hemoglobin
IP: Inappropriate Prescription
MDE: Monthly Drug Expenditure
MR: Medication-Review
MRCI: Medication Regimen Complexity Index
NSAID: Non-Steroidal Anti-Inflammatory Drugs (NSAID)
PCNE: Pharmaceutical Care Network Europe
PCP: Patient-Centered-Prescription
SBP: Systolic Blood Pressure
SD: Standard Deviation
SGLT2: Sodium-Glucose Transport Protein 2
SPSS: Statistical Package for the Social Sciences
SU: Sulphonylureas
TC: Total Cholesterol
T2DM: Type 2 Diabetes Mellitus
